# Headspace Volatile Composition of the Flowers of *Caralluma europaea* N.E.Br. (Apocynaceae)

**DOI:** 10.3390/molecules14114597

**Published:** 2009-11-11

**Authors:** Carmen Formisano, Felice Senatore, Giovanna Della Porta, Mariarosa Scognamiglio, Maurizio Bruno, Antonella Maggio, Sergio Rosselli, Pietro Zito, Maurizio Sajeva

**Affiliations:** 1 Dipartimento di Chimica delle Sostanze Naturali, Università degli Studi di Napoli “Federico II”, Via D. Montesano, 49, I-80131 Napoli, Italy; 2 Dipartimento di Ingegneria Chimica ed Alimentare, Università degli Studi di Salerno,Via Ponte Don Melillo, 84084 Fisciano (SA) Italy; 3 Dipartimento di Chimica Organica, Università degli Studi di Palermo, Viale delle Scienze, Parco d’Orleans II, I-90128 Palermo, Italy; 4 Dipartimento di Scienze Botaniche dell’Università di Palermo, Via Archirafi 38, I-90123 Palermo, Italy

**Keywords:** *Caralluma europaea*, *Apteranthes europaea*, Diptera, pollination, sapromyiophily, volatiles

## Abstract

The volatile constituents of the flowers of *Caralluma europaea* (Guss.) N.E.Br (Apocynaceae) from Lampedusa Island were analyzed by a headspace GC method. The analyses allowed the identification and quantification of 41 compounds. The main components were, among the monoterpenoids, terpinolene (23.3%), α-terpinene (19.1%) and linalool (18.4%), whereas, among the carbonylic compounds the major constituents were heptanal (2.0%), octanoic acid (2.4%) and hexanoic acid (1.7%). The presence of a nitrogen containing compound, indole (0.8%) and of a sulphur containing compound, dimethylsulphide (t), noteworthy. The compounds found in the flowers of *C. europaea* have been compared with data available in the literature as regard to their odor, presence in other sapromyiophilous taxa, possible role as semiochemicals, and presence in decaying organic matter. 89.3% of total constituents have been described in other sapromyiophilous taxa. Some of the compounds are present in several types of decaying organic matter (excrements, decomposing bodies, and spoiled fish, etc). Several volatiles found in *C. europaea* flowers are used as semiochemicals by Hymenoptera, Coleoptera, Diptera, Lepidoptera and other insects. Sixteen volatiles, accounting for 32.4% of the total constituents, are described as attractants of some Diptera families, with a biology linked to decaying organic matter. Our data thus confirm that *C. europaea* floral bouquet falls within the sapromyiophilous pollination syndrome.

## Introduction

Headspace GC analysis is widely used to study the volatiles composition of flowers and over 1,700 volatile compounds have been identified so far, as reported in a review by Knudsen *et al.* [1] describing volatiles present in the flowers of 991 taxa of the Angiosperms and a few Gymosperms. The volatile composition of flowers plays a major role, besides other tasks, in attracting insects involved in pollination [2]. Most floral scent are bouquets composed of at least a few, but usually many components. Although the blend is often dominated by one or a few main components, this does not necessarily mean that said component(s) provide the most important signal to the pollinators but instead it is the total scent [2]. Plant species with similar pollinators show similarities, not only in the visual attractants but also in the floral scent composition, regardless of the phylogenetic relatedness of the species. 

*Caralluma europaea* (Guss.) N.E.Br [= *Apteranthes europaea* (Guss.) Plowes] belongs to the family Apocynaceae, subfamily Asclepiadoideae. In this subfamily the pollination system is phenotypically high specialized, with pollen aggragated in pollinia [3] like Orchidaceae, and almost all the taxa are insect pollinated. However, while the volatile composition of the flowers of Orchidaceae has been investigated by several authors, relatively few studies have been carried out on the Apocynaceae [1]. Recently Jürgens *et al*. [4,5] analysed the chemical volatile composition of some Asclepiadoideae and discussed their possible role in the biology of pollination. The chemical composition of flowers of the genus *Caralluma* is little studied, the only available data being those reported by Jürgens *et al*. [4] for *Desmidorchis flava* (N.E.Br) Meve & Liede (= *Caralluma flava* N.E.Br) and *Apteranthes joannis* (Maire) Plowes (= *Caralluma joannis* Maire).

In the present paper we present the volatile composition of flowers of *C. europaea* as determined by headspace analysis and discuss the possible role in its pollination biology. *C. europaea*, like most Asclepiadoideae, is pollinated by Diptera and falls within the sapromyiophilous syndrome where insects are attracted by the color and odor of the flowers simulating breeding sites or food sources [4].

## Results and Discussion

In the flowers of *C. europaea* we detected 41 compounds (Table 1) belonging to nine different classes. The analyses allowed the identification of monoterpenoids, sesquiterpenoids, alcohols, aldehydes, ketones, acids and derivatives, nitrogen- and sulphur bearing compounds, and phenols. More than two thirds of the identified compounds were isoprenoids. In fact monoterpenoids were the main components, representing 77.0% of the compounds identified, accompanied by sesquiterpenoids (1.7%). Among these compounds, the monoterpene hydrocarbons were the most representative, amounting to 56.7%, with terpinolene (23,3%) and α-terpinene (19.1%) as the main components of this fraction. Linalool (18.4%) was the main oxygen containing monoterpene and represented almost the whole content of these compounds, being the remaining four compounds of this class present in lower amounts (0.2%-0.7%).

**Table 1:** LRI, RI and percent composition of the volatile compounds of the flowers of *Caralluma europaea*.

LRI^a^	Compound	%	Ident.^b^	LRI^a^	Compound	%	Ident.^b^
<800	2-Methylbutanal	1.3	R_i_, MS	1097	Linalool	18.4	R_i_, MS, P.C.
<800	Dimethyl sulphide	t	R_i_, MS	1101	Nonanal	1.0	R_i_, MS
866	Hexanol	1.1	R_i_, MS	1144	(*Z*)-Verbenol	0.4	R_i_, MS
901	Heptanal	2.0	R_i_, MS	1180	Octanoic acid	2.4	R_i_, MS, P.C.
912	Santolinatriene	2.2	R_i_, MS	1193	Myrtenal	0.6	R_i_, MS
927	Tricyclene	1.2	R_i_, MS	1194	Myrtenol	0.6	R_i_, MS
938	α-Pinene	1.9	R_i_, MS, P.C.	1196	4-Ethylbenzaldehyde	0.2	R_i_, MS
953	Camphene	2.5	R_i_, MS, P.C.	1206	Verbenone	0.7	R_i_, MS
963	Benzaldehyde	0.4	R_i_, MS	1276	3-Ethyl-4-methyl-1 *H*-pyrrole-2;5-dione	0.5	R_i_, MS
978	β-Pinene	3.8	R_i_, MS, P.C.	1278	Nonanoic acid	1.2	R_i_, MS, P.C.
980	Phenol	0.8	R_i_, MS	1290	Indole	0.8	R_i_, MS
981	Hexanoic acid	1.7	R_i_, MS, P.C.	1376	Decanoic acid	0.8	R_i_, MS
998	Octanal	0.8	R_i_, MS	1377	α-Copaene	0.3	R_i_, MS
1005	α-Phellandrene	1.4	R_i_, MS	1385	β-Bourbonene	0.2	R_i_, MS
1009	Carene 3	0.5	R_i_, MS	1398	α-Elemene	0.3	R_i_, MS
1013	α-Terpinene	19.1	R_i_, MS, P.C.	1416	(*E*)-Caryophyllene	0.6	R_i_, MS
1025	*p*-Cymene	0.8	R_i_, MS	1462	Seychellene	0.8	R_i_, MS
1037	Benzyl alcohol	0.5	R_i_, MS	1447	2;6-Di- *tert*-butyl-benzoquinone	0.2	R_i_, MS
1044	Phenylacetaldehyde	0.4	R_i_, MS	1519	1S- *cis*-Calamenene	t	R_i_, MS
1067	Octanol	0.8	R_i_, MS	1541	α-Calacorene	0.1	R_i_, MS
1086	Terpinolene	23.3	R_i_, MS, P.C.	**Total compounds 96.6%**			

^a^ LRI: linear retention indices; ^b^ Ident. R_i_: retention index; MS: mass spectrum, P.C.: same behaviour of the pure compound; t: trace amounts < 0.1%.

Phenol, indole and dimethyl sulphide were the only compounds of the benzenoid, nitrogen and sulphur bearing compounds noted, the former two being present in the same amount (0.8%), while the latter was detected only in traces. Acids and derivatives and aldehydes were present in quite similar amounts but the aldehydes are present with a greater number of compounds. 

Thirty-three compounds, accounting for 89.3% of the total constituents, have been found in several sapromyiophilous taxa (Table 2 and references therein). Besides being present in sapromyiophilous taxa some of the compounds found are present in several types of decaying organic matter. As regards to excrements, heptanal, octanal, hexanol, nonanal, benzaldehyde, phenylacetaldehyde, phenol and indole have been found in dog faeces [6], hexanol, octanol and phenol in rabbit faeces [7], indole in faeces of lion, Canidae and Mustelidae [4]. In cow dung [8] nonanal is consistently present, while phenol, benzyl alchohol, benzaldehyde and indole were erratically sampled, and are important volatiles of the fresh dung of cattle [8]. Within animals α-pinene has been detected in human bodies in decomposition [9]; nonanal, octanal and benzaldehyde in spoiled fish [10]; heptanal, phenol, nonanal, decanal and nonanoic acid in smoked salmon [11]; nonanal in human bodies in decomposition and in humans, deer and dog bones [12]; 2-methylbutanal, heptanal, hexanoic acid, octanal, nonanal, decanal nonanoic acid and indole in cooked beef meats [13]; hexanoic acid, octanoic acid and nonanoic acid, indole and phenol in pig living quarters [14]. The presence of hexanol, heptanal, nonanal, phenylacetaldehyde, indole, dimethylsulphide, α-pinene, linalool, hexanoic acid, octanoic acid, nonanoic acid and decanoic acid has been reported in various types of cheese [15]. 

As regard to the presence in other sapromyiophilous taxa, traces of terpinolene are present in *Desmidorchis flava* and in *Hoodia gordoni* (Masson) Sweet (Apocynaceae) [4], in *Arum maculatum* L. (Araceae) where it accounts for 1-10% [16], and in the club-shaped organs of *Sauromatum guttatum* (Wallich) Schott. (Araceae). with 37.9% [17]. α-Terpinene and linalool are also present in the club-shaped organs of *Sauromatum guttatum*, where they account for 0.2% and 5.5%, respectively [17], while 0.8% of linalool has been found in the pistillate-stage spate of *Peltandra virginica* (L.) Kunth (Araceae) [18], and 1.5% in flowers of *Zizyphus mauritiana* Mill. (Rhamnaceae) [19]. Hexanoic acid and octanoic acid are present in *Desmidorchis flava* and, according to Jürgens *at al*. [4], are responsible for the typical urine odor. The frequent presence of hexanoic acid in the urine of various animals seems to be caused by bacterial activity [4].

Several volatile compounds found in *C. europaea* (Table 2) are used in Hymenoptera, Coleoptera, Diptera, Lepidoptera and others insects as semiochemicals (attractants, allomones, kairomones, pheromones); and one, (*E*)-caryophyllene, is a synomone for Hymenoptera [20]. Interstingly, 16 volatile compounds of *C. europaea* flowers, accounting for 32.4% of the total constituents, are described by El-Sayed [20] as attractants of some families of Diptera. In particular linalool, the third more abundant compound in *C. europaea*, is an attractant of the families Psilidae, Muscidae, Sarcophagidae and Tephritidae [20]. Some classes of compounds, like fatty acid derivatives, sulphur- and or nitrogen- compounds, alcohols, ketones and aldehydes found in the flowers of *C. europaea* are associated with Diptera and Coleoptera which feed or ovideposit on decaying organic matter [21]. Recently Jürgens *et al*. [5] noted that most of the compounds found in 15 taxa of Apocynaceae are widespread floral scent compounds and that 84% of them are present in plants mainly pollinated by Hymenoptera and Lepidoptera, but also in 15 taxa of stapeliads with fetid floral odors [4]; the authors ascribe the presence of attractant of Lepidoptera and Hymenoptera to the phylogenetic relatedness of the plants involved.

**Table 2:** Volatile compounds of the flowers of *Caralluma europaea* arranged by class.

Compounds	Odour characteristic ^a^	Sapromyiophilous taxa ^b^	Semiochemicals^c^				
			A	Al	P	K	Sy
**Alchools**							
Octanol	Metallic; sulfur; burnt matches; toasted bread; herbal; fatty; floral; woody; citrus; waxy; moss; nut; mushroom	*Hoodia currori* (Hook.) Decne (Apocynaceae) [4]	x	x	x	-	-
		*Hoodia gordonii* (Masson) Sweet (Apocynaceae) [4]					
		*Huernia boleana* M.G. Gilbert (Apocynaceae) [4]					
		*Huernia keniensis* R.E. Fries (Apocynaceae) [4]					
		*Monolluma exagona* (Lavranos) Meve & Liede (Apocynaceae) [4]					
		*Orbea semota* (N.E.Br) L.C. Leach subsp. *orientalis* Bruyns (Apocynaceae) [4]					
		*Orbea variegata* (L.) L.C. Leach (Apocynaceae) [4]					
		*Stapelia asterias* Masson (Apocynaceae) [4]					
		*Sauromatum guttatum* (Wallich) Schott. (Araceae) [22]					
Benzyl alcohol	Berry; cherry; grapefruit; citrus; walnut; sweet	*Duvalia corderoyi* N.E.Br (Apocynaceae) [4]	x	x	x	x	-
		*Hoodia currori* (Hook.) Decne (Apocynaceae) [4]					
		*Hoodia gordonii* (Masson) Sweet (Apocynaceae) [4]					
		*Monolluma exagona* (Lavranos) Meve & Liede (Apocynaceae) [4]					
		*Orbea semota* (N.E.Br) L.C. Leach subsp*. orientalis* Bruyns					
		(Apocynaceae) [4]					
		*Orbea variegata* (L.) L.C. Leach (Apocynaceae) [4]					
		*Stapelia asterias* Masson (Apocynaceae) [4]					
		*Arum creticum* Boiss. & Heldr. (Araceae) [16-23]					
		*Arum idaeum* Coust. & Gand. (Araceae) [23]					
		*Sauromatum guttatum* (Wallich) Schott. (Araceae) [17-22]					
		*Zizyphus mauritiana* Mill*.* (Rhamnaceae) [19 ]					
Hexanol	Flowery; toasty; dry; fruity; herbal; mild woody; sweet; green grass; leafy; resin	*Hoodia currori* (Hook.) Decne (Apocynaceae) [4]	x	x	x	x	-
*Sauromatum guttatum* (Wallich) Schott. (Araceae) [17]							
**Aldehydes **							
2-Methylbutanal	-	-	-	-	-	-	-
Heptanal	Citrus fruit; green; fatty; dry fish; pesticide; solvent; smoky; rancid; fruity; oily; woody; fruity; nutty; heavy; putty; soapy	*Desmidorchis* *flava* (N.E.Br) Meve & Liede (Apocynaceae) [4]	x	x	x	x	-
		*Echidnopsis montana* (R.A. Dyer & E.A. Bruce) P.R.O. Bally (Apocynaceae) [4]					
		*Hoodia gordonii* (Masson) Sweet (Apocynaceae) [4]					
		*Huernia boleana* M.G. Gilbert (Apocynaceae) [4]					
		*Huernia keniensis* R.E. Fries (Apocynaceae) [4]					
		*Monolluma exagona* (Lavranos) Meve & Liede (Apocynaceae) [4]					
		*Orbea semota* (N.E.Br) L.C. Leach subsp*. orientalis* Bruyns(Apocynaceae) [4]					
		*Orbea variegata* (L.) L.C. Leach (Apocynaceae) [4]					
		*Stapelia asterias* Masson (Apocynaceae) [4]					
		*Hydnora africana* Thunb. (Hydnoraceae) [1]					
		*Zizyphus mauritiana* Mill*.*(Rhamnaceae) [19]					
Benzaldehyde	Burnt sugar; almond; woody; cherry; sweet	*Apteranthes joannis* (Maire) Plowes (Apocynaceae) [4]	x	x	x	x	-
		*Desmidorchis flava* (N.E.Br) Meve & Liede (Apocynaceae) [4]					
		*Echidnopsis leachii* Lavranos (Apocynaceae) [4]					
		*Echidnopsis montana* (R.A. Dyer & E.A. Bruce) P.R.O. Bally(Apocynaceae) [4]					
		*Hoodia currori* (Hook.) Decne (Apocynaceae) [4]					
		*Hoodia gordonii* (Masson) Sweet (Apocynaceae) [4]					
		*Huernia boleana* M.G. Gilbert (Apocynaceae) [4]					
		*Huernia keniensis* R.E. Fries (Apocynaceae) [4]					
		*Monolluma exagona* (Lavranos) Meve & Liede (Apocynaceae) [4]					
		*Orbea semota* (N.E.Br) L.C. Leach subsp*. orientalis* Bruyns(Apocynaceae) [4]					
		*Orbea variegata* (L.) L.C. Leach (Apocynaceae) [4]					
		*Pseudolithos cubiformis* (P.R.O. Bally) P.R.O. Bally (Apocynaceae) [4]					
		*Stapelia asterias* Masson (Apocynaceae) [4]					
		*Arisaema erubescens* (Wallich) Schott. (Araceae)^24^					
		*Arum creticum* Boiss. & Heldr. (Araceae) [16-23]					
		*Arum idaeum* Coust. & Gand*.* (Araceae)^23^					
		*Sauromatum guttatum* (Wallich) Schott. (Araceae) [17]					
		*Hydnora africana* Thunb*.* (Hydnoraceae) [1]					
		*Zizyphus mauritiana* Mill (Rhamnaceae) [19]					
Octanal	Lemon; stew-like; boiled meat-like; rancid; soapy; citrus; green; flower; fruit; orange; honey; fatty; pungent; slightly fragment	*Apteranthes joannis* (Maire) Plowes (Apocynaceae) [4]	x	x	x	x	-
		*Echidnopsis leachii* Lavranos (Apocynaceae) [4]					
		*Echidnopsis montana* (R.A. Dyer & E.A. Bruce) P.R.O. Bally (Apocynaceae) [4]					
		*Hoodia currori* (Hook.) Decne (Apocynaceae) [4]					
		*Hoodia gordonii* (Masson) Sweet (Apocynaceae) [4]					
		*Huernia boleana* M.G. Gilbert (Apocynaceae) [4]					
		*Huernia keniensis* R.E. Fries (Apocynaceae)[4]					
		*Monolluma exagona* (Lavranos) Meve & Liede (Apocynaceae)[4]					
		*Orbea semota* (N.E.Br) L.C. Leach subsp*. orientalis* Bruyns(Apocynaceae)[4]					
		*Orbea variegata* (L.) L.C. Leach (Apocynaceae)[4]					
		*Pseudolithos cubiformis* (P.R.O. Bally) P.R.O. Bally (Apocynaceae)[4]					
		*Stapelia asterias* Masson (Apocynaceae)[4]					
		*Arisaema erubescens* (Wallich) Schott*.*(Araceae) [24]					
		*Sauromatum* guttatum (Wallich) Schott*.* (Araceae) [22]					
		*Hydnora africana* Thunb*.* (Hydnoraceae) [1]					
		*Zizyphus mauritiana* Mill*.* (Rhamnaceae) [19]					
Phenylacetaldehyde	Apple; apricot; berry; cherry; chocolate; grape; grapefruit; honey; hyacinth; lemon; melon; orange; green; nutty; fruity; peach; peanut; vegetable; winelike; sweet; honey like; flower; daisy	*Hoodia currori* (Hook.) Decne (Apocynaceae)[4]	-	-	-	-	-
		*Hoodia gordonii* (Masson) Sweet (Apocynaceae)[4]					
		*Huernia boleana* M.G. Gilbert (Apocynaceae)[4]					
		*Huernia keniensis* R.E. Fries (Apocynaceae)[4]					
		*Orbea variegata* (L.) L.C. Leach (Apocynaceae)[4]					
		*Stapelia asterias* Masson (Apocynaceae)[4]					
Nonanal	Gravy; green; tallowy; fruity; gas; chlorine; floral; waxy; sweet; melon; soapy; fatty; lavender; citrus fruit; apple; coconut; grape; grapefruit; lemon; lime; oily; orange; waxy; citrus; fatty; nutty; peach; rose; vegetable; meaty; fishy; slightly pungent; grass-like; animals	*Arisaema ciliatum* H. Li (Araceae)^2^[4]	x	x	x	x	-
		*Arisaema erubescens* (Wallich) Schott*.* (Araceae)^24^					
		*Arisaema lobatum* Engl. (Araceae) [24]					
		*Arisaema tortuosum* (Wallich) Schott*.* (Araceae)[24]					
		*Arum idaeum* Coust. & Gand*.* (Araceae) [23]					
		*Arum maculatum* L. (Araceae)^8^					
		*Peltandra virginica* (L.) Kunth. (Araceae) [18]					
		*Sauromatum guttatum* (Wallich) Schott. (Araceae) [22]					
		*Hydnora africana* Thunb*.* (Hydnoraceae) [1]					
		*Zizyphus mauritiana* Mill*.* (Rhamnaceae) [19]					
4-Ethylbenzaldehyde	Fruity; anisic; minty; balsamic-sweet; nutty-almond	*Hoodia gordonii* (Masson) Sweet (Apocynaceae)[4]	-	-	-	-	-
**Ketones**							
3-Ethyl-4-methyl-1*H*-pyrrole-2;5-dione	-	-	-	-	-	-	-
2;6-Di-*tert*-butylbenzoquinone	-	-	-	-	-	-	-
**Nitrogen containg compounds**							
Indole	Butter; cheese; chocolate; grape; honey; jasmine; musty; floral; fatty; vanilla; animal; earthy; vegetable; wine-like; fishy; musty fecal; mothball-like	*Apteranthes joannis* (Maire) Plowes (Apocynaceae)[4]	x	x	x	x	-
		*Hoodia gordonii* (Masson) Sweet (Apocynaceae)[4]					
		*Orbea variegata* (L.) L.C. Leach (Apocynaceae)[4]					
		*Amorphophallus eichleri* (Engl.) Hook. f. (Araceae)[25]					
		*Arum apulum* (Carano) P.C. Boyce (Araceae) [16]					
		*Arum cyrenaicum* Hruby (Araceae) [16]					
		*Arum creticum* Boiss. & Heldr*.* (Araceae)[16]					
		*Arum maculatum* L*.* (Araceae) [8,9,^10^,^11^,^12^,^13^,^14^,^15^,^16^]					
		*Arum nigrum* Schott. (Araceae) [16]					
		*Sauromatum guttatum* (Wallich) Schott. (Araceae) [17,18,19,20,21,22]					
**Acids and derivatives**							
Hexanoic acid	Sweaty; pungent; cheese; goatlike; rancid; fatty; sour; bad breath; popcorn; goaty	*Desmidorchis flava* (N.E.Br) Meve & Liede (Apocynaceae)[4]	x	x	x	x	-
		*Duvalia corderoyi* N.E.Br (Apocynaceae)[4]					
		*Echidnopsis montana* (R.A. Dyer & E.A. Bruce) P.R.O. Bally(Apocynaceae)[4]					
		*Hoodia currori* (Hook.) Decne (Apocynaceae)[4]					
		*Hoodia gordonii* (Masson) Sweet (Apocynaceae)[4]					
		*Orbea variegata* (L.) L.C. Leach (Apocynaceae)[4]					
		*Stapelia asterias* Masson (Apocynaceae)[4]					
		*Sauromatum guttatum* (Wallich) Schott. (Araceae) [17]					

Octanoic acid	Fatty acid; cheese; fresh; moss; oily; body odour; rancid, pungent; sweet	*Desmidorchis flava* (N.E.Br) Meve & Liede (Apocynaceae)[4]	-	x	x	x	-
Nonanoic acid	Green; fat; musty; sweaty; sour; cheese; waxy; goat	*Apteranthes joannis* (Maire) Plowes (Apocynaceae)[4]	x	x	x	-	-
		*Desmidorchis* *flava* (N.E.Br) Meve & Liede (Apocynaceae)[4]					
		*Duvalia corderoyi* N.E.Br (Apocynaceae)[4]					
		*Hoodia currori* (Hook.) Decne (Apocynaceae)[4]					
		*Hoodia gordonii* (Masson) Sweet (Apocynaceae)[4]					
		*Huernia keniensis* R.E. Fries (Apocynaceae)[4]					
		*Orbea variegata* (L.) L.C. Leach (Apocynaceae)[4]					
		*Stapelia asterias* Masson (Apocynaceae)[4]					
		*Sauromatum guttatum* (Wallich)					
		Schott. (Araceae) [17,18,19,20,21,22]					
		*Zizyphus mauritiana* Mill. (Rhamnaceae) [19]					
Decanoic acid	Soapy; Fatty; citrus; warm; butter; fruit; grassy; cheese; milk; rancid	*Apteranthes joannis* (Maire) Plowes (Apocynaceae) [4]	-	x	x	-	-
		*Desmidorchis flava* (N.E.Br) Meve & Liede (Apocynaceae)[4]					
		*Echidnopsis leachii* Lavranos (Apocynaceae)[4]					
		*Hoodia currori* (Hook.) Decne (Apocynaceae)[4]					
		*Hoodia gordonii* (Masson) Sweet (Apocynaceae)[4]					
		*Huernia boleana* M.G. Gilbert (Apocynaceae)[4]					
		*Huernia keniensis* R.E. Fries (Apocynaceae)[4]					
		*Orbea semota* (N.E.Br) L.C. Leach subsp*. orientalis* Bruyns (Apocynaceae)[4]					
		*Orbea variegata* (L.) L.C. Leach (Apocynaceae)[4]					
		*Stapelia asterias* Masson (Apocynaceae)[4]					
		*Sauromatum guttatum* (Wallich) Schott. (Araceae)^22^					
**Monoterpenoids**							
Santolinatriene	-	-	-	-	-	-	-
Tricyclene	-	-	-	-	-	-	-
α-Pinene	Terpeny; fruity; sweet; green; woody; pine; citrus; lime; camphoraceous; turpentine	*Desmidorchis flava* (N.E.Br) Meve & Liede (Apocynaceae)[4]	x	x	x	x	-
		*Echidnopsis leachii* Lavranos (Apocynaceae)[4]					
		*Echidnopsis montana* (R.A. Dyer & E.A. Bruce) P.R.O. Bally (Apocynaceae)[4]					
		*Hoodia currori* (Hook.) Decne (Apocynaceae)[4]					
		*Hoodia gordonii* (Masson) Sweet (Apocynaceae)[4]					
		*Huernia keniensis* R.E. Fries (Apocynaceae)[4]					
		*Arum maculatum* L. (Araceae) [8]					
		*Peltandra virginica* (L.) Kunth. (Araceae) [18]					
		*Sauromatum guttatum* (Wallich) Schott. (Araceae) [17,18,19,20,21,22]					
Camphene	Sweet; fruity; camphoraceous; pine; oily; herbal; vanilla	*Sauromatum guttatum* (Wallich) Schott. (Araceae) [17-22]	x	x	x	x	-
β-Pinene	Musty; green; sweet; pine; resin; turpentine; woody	*Arum maculatum* L*.* (Araceae)^8-16^	x	x	x	x	-
		*Peltandra virginica* (L.) Kunth. (Araceae)[18]					
		*Sauromatum guttatum* (Wallich) Schott. (Araceae)[17]					
α-Phellandrene	Fruity; minty; herbaceous; citrus; lime; pepper; juniper; dill	*Sauromatum guttatum* (Wallich) Schott. (Araceae) [17,18,19,20,21,22]	x	x	x	-	-
Carene 3	Citrus fruit; orange peel; lemon	*Apteranthes joannis* (Maire) Plowes (Apocynaceae)[4]	x	x	x	x	-
		*Echidnopsis leachii* Lavranos (Apocynaceae)[4]					
		*Echidnopsis montana* (R.A. Dyer & E.A. Bruce) P.R.O. Bally (Apocynaceae)[4]					
		*Hoodia currori* (Hook.) Decne (Apocynaceae)[4]					
		*Hoodia gordonii* (Masson) Sweet (Apocynaceae) [4]					
		*Huernia boleana* M.G. Gilbert (Apocynaceae) [4]					
		*Huernia keniensis* R.E. Fries (Apocynaceae) [4]					
		*Orbea semota* (N.E.Br) L.C. Leach subsp*. orientalis* Bruyns (Apocynaceae) [4]					
		*Orbea variegata* (L.) L.C. Leach (Apocynaceae) [4]					
		*Peltandra virginica* (L.) Kunth. (Araceae)[18]					
		*Sauromatum guttatum* (Wallich) Schott. (Araceae) [17]					
α-Terpinene	Gasoline-like; ethereal; fruity; lemon; berry; sweet; vegetable; woody; pepper; medicinal; camphoraceous	*Sauromatum guttatum* (Wallich) Schott. (Araceae) [17,18,19,20,21,22]	-	x	x	x	-
*p*-Cymene	Lemon; fruity; fuel-like; sweet; herbal; spicy; citrus; solvent; gasoline	*Apteranthes joannis* (Maire) Plowes (Apocynaceae) [4]	x	x	x	x	-
*Sauromatum guttatum* (Wallich) Schott. (Araceae) [17-22]							
Terpinolene	Woody; fruity; sweet; piney; slightly anisic; plastic	*Desmidorchis flava* (N.E.Br) Meve & Liede (Apocynaceae) [4]	x	x	x	x	-
		*Hoodia gordonii* (Masson) Sweet (Apocynaceae) [4]					
		*Arum maculatum* L*.* (Araceae) [8-16]					
		*Sauromatum guttatum* (Wallich) Schott. (Araceae) [22]					
Linalool	Lemon; orange; citrus; floral; sweet; aniseed; lavender, muscat, parsley, fruity	*Peltandra virginica* (L.) Kunth. (Araceae) [18]	x	x	x	x	-
		*Sauromatum guttatum* (Wallich) Schott. (Araceae) [17,18,19,20,21,22]					
		*Zizyphus mauritiana* Mill. (Rhamnaceae) [19]					
(*Z*)-Verbenol	-	-	-	-	-	-	-
Myrtenal	Sweet; sharp; pleasant; spicy; cinnamon	-	x	-	-	-	-
Myrtenol	Medicinal; berry; medicinal; minty; woody; vanilla	*Sauromatum guttatum* (Wallich) Schott. (Araceae) [22]	x	-	x	x	-
		*Zizyphus mauritiana* Mill. (Rhamnaceae) [19]					

Verbenone	Minty; spicy	*Arisaema tortuosum* (Wallich) Schott. (Araceae) [1-24]	x	x	x	x	-
**Sesquiterpenoids**			**	**	**	**	**
α-Copaene	Woody; earthy; spicy	*Desmidorchis flava* (N.E.Br) Meve & Liede (Apocynaceae) [4]	x	-	x	-	-
		*Echidnopsis leachii* Lavranos (Apocynaceae) [4]					
		*Hoodia gordonii* (Masson) Sweet (Apocynaceae) [4]					
		*Huernia keniensis* R.E. Fries (Apocynaceae) [4]					
		*Arum maculatum* L*.* (Araceae) [8]					
		*Sauromatum guttatum* (Wallich) Schott. (Araceae) [17,18,19,20,21,22]					
β-Bourbonene	Herbaceous	*Hoodia gordonii* (Masson) Sweet (Apocynaceae) [4]	-	-	x	-	-
α-Elemene	-	-	-	-	-	-	-
(*E*)-Caryophyllene	Musty; green; spicy; woody; terpene-like; fruity; sweet	*Desmidorchis flava* (N.E.Br) Meve & Liede (Apocynaceae) [4]	x	x	x	x	x
		*Hoodia gordonii* (Masson) Sweet (Apocynaceae) [4]					
		*Monolluma exagona* (Lavranos) Meve & Liede (Apocynaceae) [4]					
		*Orbea semota* (N.E.Br) L.C. Leach subsp*. orientalis* Bruyns (Apocynaceae) [4]					
Seychellene	-	*Desmidorchis flava* (N.E.Br) Meve & Liede (Apocynaceae) [4]	-	-	-	-	-
*Hoodia gordonii* (Masson) Sweet (Apocynaceae) [4]							
1S-*cis*-Calamenene	-	*Desmidorchis flava* (N.E.Br) Meve & Liede (Apocynaceae) [4]	-	-	-	-	-
		*Hoodia gordonii* (Masson) Sweet (Apocynaceae) [4]					
		*Huernia keniensis* R.E. Fries (Apocynaceae) [4]					
		*Sauromatum guttatum* (Wallich) Schott. (Araceae) [22]					
α-Calacorene	Dry-woody	*Sauromatum guttatum* (Wallich) Schott. (Araceae) [17]	-	-	-	-	-
**Phenols**							
Phenol	Phenolic; medicinal odor	*Duvalia corderoyi* N.E.Br (Apocynaceae) [4]	x	x	x	x	-
		*Hoodia gordonii* (Masson) Sweet (Apocynaceae) [4]					
		*Hoodia currori* (Hook.) Decne (Apocynaceae) [4]					
		*Monolluma exagona* (Lavranos) Meve & Liede (Apocynaceae) [4]					
		*Orbea semota* (N.E.Br) L.C. Leach subsp*. orientalis* Bruyns (Apocynaceae) [4]					
		*Orbea variegata* (L.) L.C. Leach (Apocynaceae) [4]					
		*Sauromatum guttatum* (Wallich) Schott. (Araceae) [22]					
**Sulphur containing compouds**							
Dimethyl sulphide	Asparagus; cabbage; corn; cowy; molasses; reminiscent of wild radish; sharp; sickly; sulfurous; vegetable; gasoline	*Arisaema tortuosum* (Wallich) Schott. (Araceae) [24]	-	-	-	-	-

^a^ Odor characteristics are taken from the following sources: [15,20,26,27,28,29]. ^b^ Sapromyiophilous taxa are taken from references quoted at the apices of reported taxa. ^c^Semiochemicals: (A: Attractant; Al: Allomone; K: Kairomone; P: Pheromone; Sy: Synomone) [20].

The volatiles found give the floral bouquet of *C. europaea* sulfur, mushroom, woody, sweet, fish, smoky, rancid, woody, pungent, fecal, cheese and other odors (Table 1 and references therein). In the literature there are several reports about the pollinators of the genus *Caralluma* indicating that this genus is pollinated by flies [30,31,32,33] but there are few data on the volatiles of the flowers. 

Flowers in taxa of the genus *Caralluma* show decaying organic matter odors, like *C. arachnoidea* with scents of rotting fruits and are pollinated by small Drosophilidae or Milichiidae, while the floral odor of *Desmidorchis flava* can be described as reminiscent of decaying urine or pungent and urinous and Coleoptera (Dermestidae) have been recorded as flower visitors, although it has not been determined whether they really act as pollinators [4]. Meve and Heneidak [3] state that *C. europaea* flowers have an odor of excrement without reporting any chemical analysis. Interstingly, 23 compounds found in *C. europaea* are also present in at least one fetid Asclepiadoideae [4], while 18 compounds are absent.

**Figure 1:**
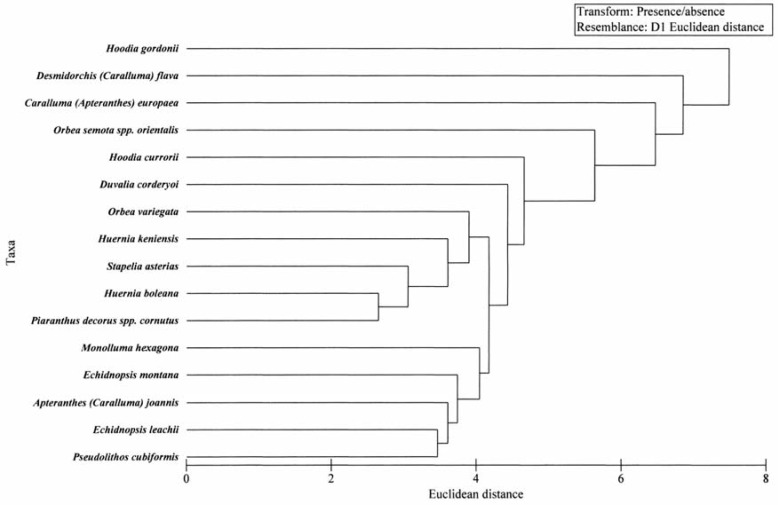
Cluster analysis based on a binary presence-absence matrix of the volatile profile of 15 taxa [4] and *C. europaea* using Euclidean distances between taxa. Unidenntified compounds were omitted from the analysis. By comparing our data with those of the 15 taxa studied by Jürgens [4] it is clear that *C. europaea* falls within the group of other three species: *Hoodia gordonii*, *Desmidorchis flava* and *Orbea semota* spp. *orientalis.*

The analysis of the volatile composition of *C. europaea* (Fig.1) combined with that of the taxa studied by Jürgens *et al*. [4] shows that twelve taxa share similar volatile composition and that *C. europaea* has similarity to the volatiles present in *Hoodia gordonii*, *Desmidorchis flava* and *Orbea semota* spp. *orientalis*. Figure 1

**Figure 2:**
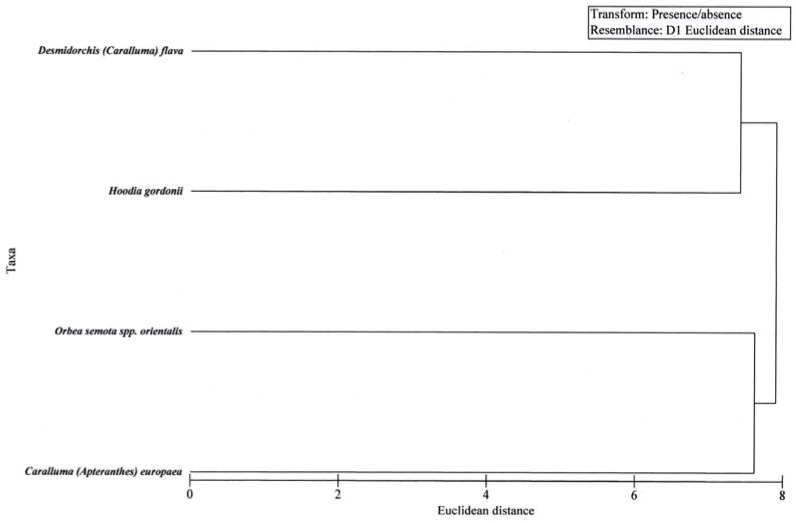
Cluster analysis based on a binary presence-absence matrix of the volatile profile of *Hoodia gordonii*, *Desmidorchis flava* and *Orbea semota* spp. *orientalis* [4] and *C. europaea* using Euclidean distances between taxa showing the close relationship of volatiles composition among the taxa.

The cluster analysis of latter group (Figure 2) confirms the similarity in volatiles of the four taxa.

## Experimental

### General

Flowers of *C. europaea* were collected in Lampedusa Island (Italy, 35°29’28” and 35°21’39” N - 12°30’54” and 12°37’55” E) in April 2009 from plants growing in the “Guitgia” area at an altitude of 20 m a.s.l. Clones of the plants are cultivated at the Botanical Garden of Palermo and a voucher specimen (N° PAL/MS/1119) was deposited in the Herbarium, Orto Botanico, Palermo, Italy. The flowers were removed from the plants with a single stroke of a razor blade, the cut surface was sealed with a drop of metacrylate (Attak^®^) to avoid the release of volatile compounds due to the cutting, placed in 20 mL autosampler vials with cripto caps and stored at –10 °C. The direct headspace analyses were performed after equilibrating the vials in a heated block. Headspace conditions were: equilibration time 35 min at 105 °C, pressurization time 1.0 min and loop fill time 1.0 min. The chemical composition was determined by using a HP 7694E headspace sampler coupled to a gas chromatograph interfaced with a HP 6890 GC SYSTEM flame ionization detector. Components were separated using two fused-silica capillary columns connected in series by press-fit: first column Carbowax EASYSEP connected to the detector, 30 m × 0.53 mm i.d., 1 µm film thickness and the second CP Sil 5CB connected to the injector; 25 m × 0.53 mm i.d., 5 µm film tickness. GLC conditions were: oven temperature 40°C with 8 min initial hold and then two ramps: the first from 40 °C to 150 °C at 2 °C/min and the second from 150 °C to 210 °C at 35 °C/min (6 min). The injector was maintained at 250 °C (splitless mode) and He was used as carrier gas (5 mL/min). Most constituents were identified by comparison of their retention indices (R_i_) with either those of the literature [34,35] or with those of authentic compounds available in our laboratories. The retention indices were determined in relation to a homologous series of *n*-alkanes (C_8_-C_18_) under the same operating conditions. Further identification was made by comparison of their mass spectra with either those stored in NIST 98 library or with mass spectra from the literature [34,36] and a home-made library. Pure commercial essential oil components used as standards for GC-FID analyses were obtained from Aldrich and Fluka. The comparison of the volatile composition of *C. europaea* with that of other taxa was performed by cluster analysis based on a binary presence-absence matrix of the volatile profile using Euclidean distances among taxa [37].

**Figure 3:**
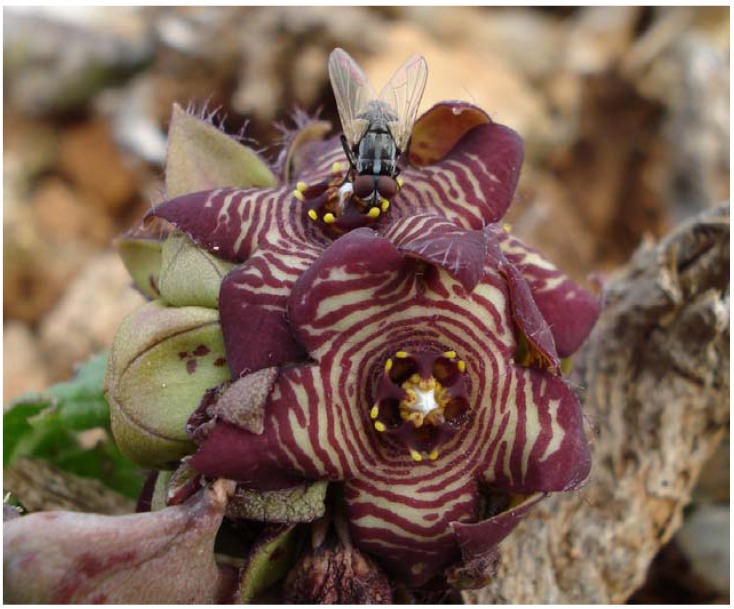
The scent of *C. europaea* flowers attracts different families of Diptera. The photo shows *Musca autumnalis* (Muscidae) inserting its proboscid in the anther slit of the flower of *C. europaea* in search of food resource. Linalool, the third more abundant compound of *C. europaea* flower volatiles, is an attractant of Muscidae (Photo by P. Zito).

## Conclusions

The volatile compounds found in the flowers of *C. europaea* seems to be a very attractive spectrum for the flies involved in pollination of this species. Flies are almost ubiquitous insects, occurring also in unfavourable habitats where other insects may be rare. Enabled by a highly sensitive olfaction system, flies are attracted to odors over long distances. *C. europaea* grows at the base of others plants or rocks or camouflaged in its environment, and flies may be the most abundant insects and therefore the sapromyiophilous syndrome reflects the adaptation of this species to this environment. Recently Pisciotta *et al.* [33] found that *C. europaea* in Lampedusa Island is pollinated by eight species of Diptera belonging to five families: Calliphoridae, Milichiidae, Muscidae (Figure 3), Sarcophagidae, and Trixoscelididae. It is interesting to note that all the Diptera families involved in pollination have a biology linked to decaying organic matters and therefore *C. europaea* falls within the sapromyiophilous pollination syndrome. According to Dobson [21] a single fly species may visit distinct flowers for different purposes (i.e. food versus oviposition) and therefore pollinate flowers. In particular flies prefer yellow in the presence of sweet scents, which signal food sources, and brown-purple in the presence of odor of excrements, which indicate egg-laying sites. Flowers of *C. europaea* are brown-purple with yellow stripes, they contain compounds with both sweet odors and compounds found in excrements, and in this way they may mimic both food resources and oviposition sites thus augmenting the spectrum of potential pollinators.
